# Pressure‐Tuned Superconducting Dome and Persistent Charge‐Density‐Wave Order in NdSeTe_2_

**DOI:** 10.1002/advs.77597

**Published:** 2026-09-03

**Authors:** Shuyang Wang, Chao An, Jiaqi Chen, Lili Zhang, Yonghui Zhou, Xuliang Chen, Zhaorong Yang

**Affiliations:** ^1^ Anhui Provincial Key Laboratory of Low‐Energy Quantum Materials and Devices High Magnetic Field Laboratory HFIPS Chinese Academy of Sciences Hefei Anhui China; ^2^ Institutes of Physical Science and Information Technology Anhui University Hefei Anhui China; ^3^ Shanghai Synchrotron Radiation Facility Shanghai Advanced Research Institute Chinese Academy of Sciences Shanghai China

**Keywords:** charge‐density‐wave, high pressure, rare‐earth tritelluride, superconductivity

## Abstract

In many charge‐density‐wave (CDW) materials, superconductivity emerges as CDW order collapses, producing a dome‐shaped phase diagram commonly associated with quantum criticality or direct competition between the two orders. Here, a distinct pressure‐tuned case is identified in NdSeTe_2_, where the superconducting dome is fully embedded within a persistent CDW state. Electrical resistivity measurements reveal a conventional superconducting dome with a maximum transition temperature of *T*_C_ ≈ 1.8 K near *P*_C_ ≈ 2.6 GPa, where long‐range CDW signatures disappear from transport. In striking contrast, high‐pressure, low‐temperature Raman spectroscopy uncovers short‐range CDW correlations that remain hidden to resistivity, emerge beyond *P*_C_, and persist up to ∼10.0 GPa, spanning the entire superconducting dome. Synchrotron X‐ray diffraction detects no structural transition up to 32.4 GPa, indicating that the CDW evolution is electronic in origin. These results establish an unusual phase diagram in which superconductivity develops within a persistent short‐range CDW background, offering new insight into pressure‐tuned collective states in low‐dimensional quantum materials.

## Graphical abstract

NdSeTe_2_ exhibits an unusual pressure‐tuned phase diagram in which charge‐density‐wave order does not disappear at optimal superconductivity. Instead, Raman spectroscopy reveals a transformation from long‐range to short‐range correlations that remain invisible to resistivity and persist across the entire superconducting dome. These findings identify persistent short‐range charge order as a key ingredient in low‐dimensional quantum materials exhibiting a full superconducting dome.

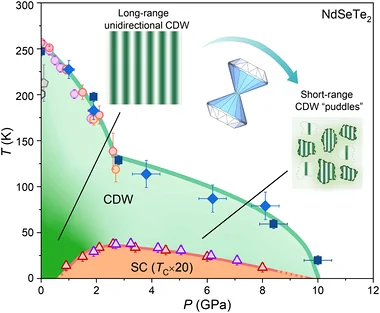

## 1 Introduction

**FIGURE 1:**
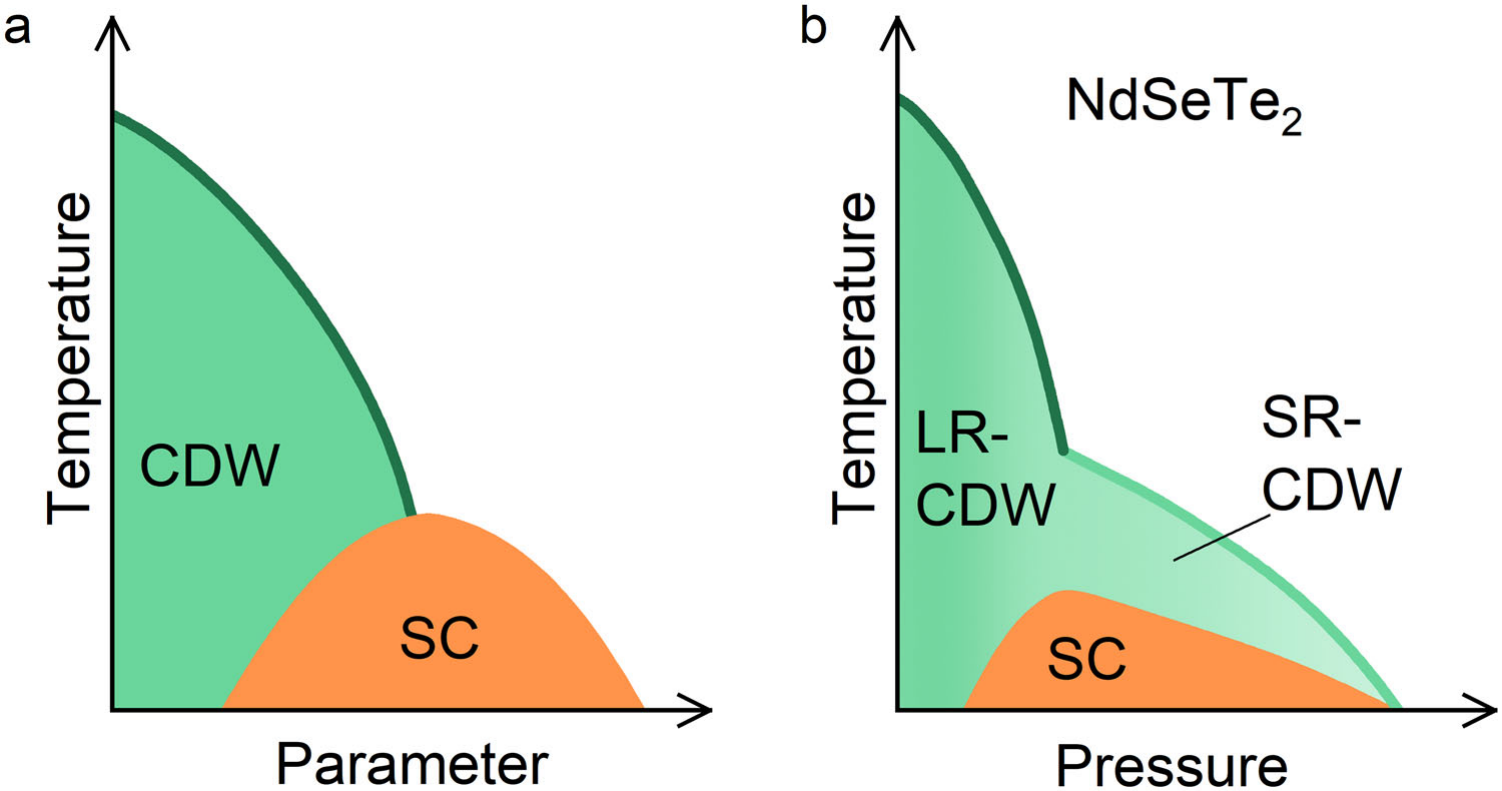
Schematic of conventional and revised CDW‐superconductivity phase diagrams. (a) A common temperature‐parameter phase diagram of prototypical CDW materials, where a superconducting dome emerges near the critical parameter point at which CDW order is suppressed–a view drawn solely from transport. (b) Schematic temperature‐pressure phase diagram of NdSeTe_2_, where the superconducting dome is fully embedded within a CDW state that evolves continuously from long‐range (LR‐CDW) to short‐range (SR‐CDW) order, established through combined transport and Raman spectroscopy. SC in (a) and (b) denotes superconductivity.

Charge‐density‐wave (CDW) order and superconductivity are often intertwined, and understanding their complex interplay remains a central issue in condensed matter physics. Constructing a complete phase diagram is essential for elucidating the concurrent evolution of CDW and superconductivity under external tuning parameters such as doping or pressure, thereby offering key insight into the mechanisms governing their interplay. In many prototypical CDW systems, such as transition‐metal dichalcogenides [1, 2, 3, 4, 5, 6], transition‐metal trichalcogenides [7, 8], LaAuSb_2_ [9], and LaPt_2_Si_2_ [10], etc., superconductivity commonly forms a dome centered near the critical parameter where CDW order collapses (Figure 1a). This trend is typically attributed either to a CDW quantum critical point beneath the dome or to direct competition between CDW order and superconductivity. However, these phase diagrams without exception are constructed solely from electrical transport measurements, which are insufficient to capture the full character of CDW order. Because a CDW involves both a periodic modulation of the electronic charge density and an accompanying lattice distortion [11], its complete understanding requires probing both the electronic and lattice degrees of freedom. Consequently, any conclusion regarding an intrinsic relationship between CDW and superconductivity remains incomplete without complementary, yet complete, information on the evolution of the CDW order itself.

A representative example is the layered CDW compound TiSe_2_. Early electrical transport studies revealed a dome‐shaped superconducting phase diagram when the CDW order was suppressed by pressure [1] or Cu intercalation [2]. The maximum superconducting transition temperature was found near a putative quantum critical point extrapolated from the vanishing CDW transition, leading to the proposal that CDW quantum fluctuations might mediate superconductivity pairing. However, subsequent X‐ray scattering experiments on pressurized TiSe_2_ [12] and X‐ray diffraction and scanning tunneling microscopy on Cu‐intercalated TiSe_2_ [13, 14, 15] revealed an intermediate regime in which pristine commensurate and emergent incommensurate CDW orders coexist near the superconducting dome. These observations, enabled by combining electrical transport with probes sensitive to CDW‐related lattice distortions, indicate that superconductivity may be linked more closely to the presence of incommensurate CDW order and associated domain walls than to critical CDW fluctuations [12, 13, 14, 16].

Quasi‐two‐dimensional rare‐earth tritellurides (RTe_3_) are well known for their unidirectional (striped) and/or bidirectional (checkerboard) CDW orders [17]. These compounds comprise two nearly square Te layers separated by an R‐Te slab, with the CDW instabilities originating in the planar Te nets [18, 19]. In heavy rare‐earth members (R = Gd, Tb, Dy, and Tm), external pressure suppresses the CDW order and can induce superconductivity at low temperatures, making this family a valuable platform for studying the interplay of CDW and superconductivity [20, 21]. However, the full superconducting phase diagram still remains experimentally unresolved, largely because previously studied single crystals usually contain extrinsic Te residues [20] and elemental Te itself becomes superconducting under high pressure [22, 23].

**FIGURE 2:**
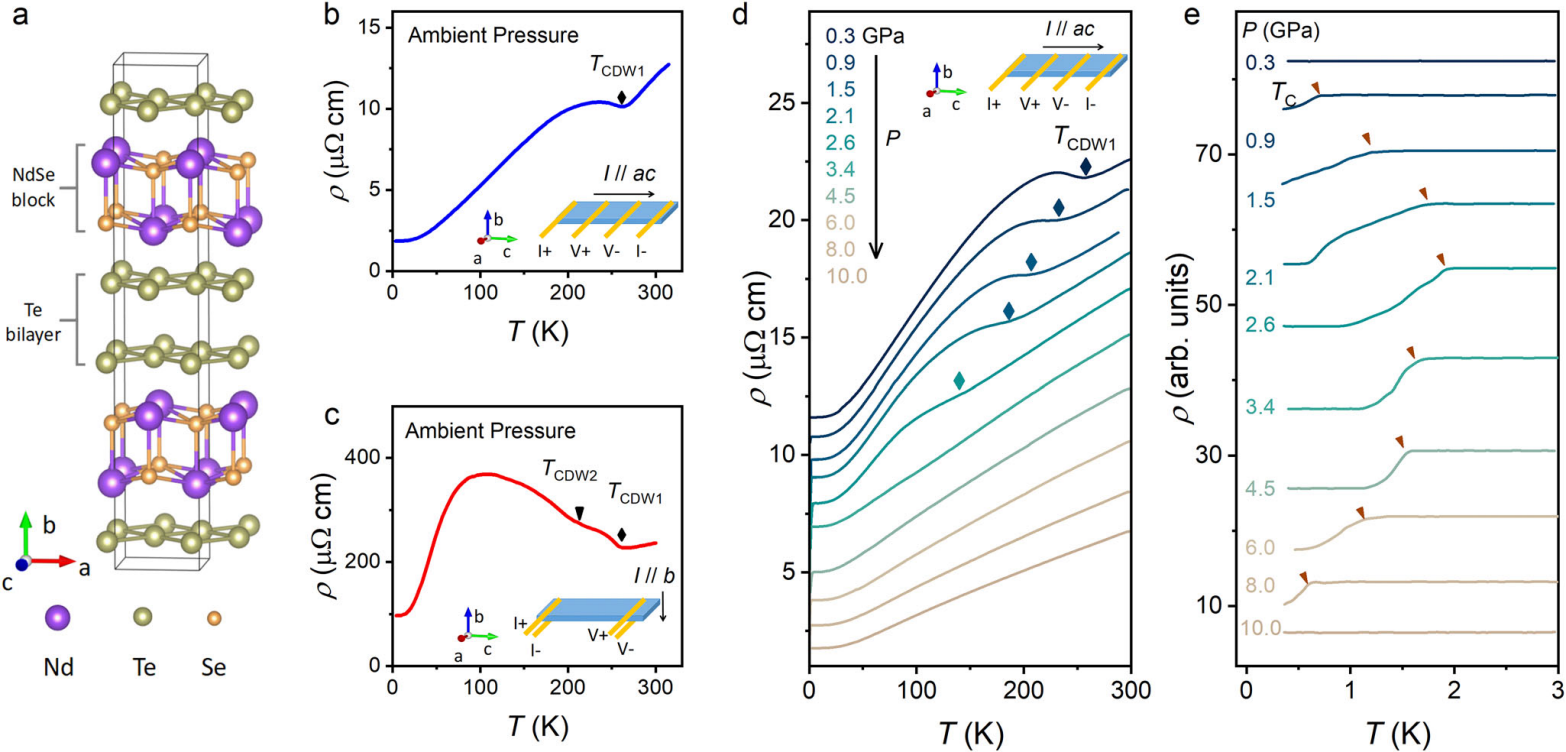
Temperature‐dependent resistivity of NdSeTe_2_ under high pressure. (a) Schematic crystal structure of NdSeTe_2_. (b) and (c) Temperature dependence of resistivity *ρ*(*T*) at ambient pressure for current applied along the *ac* plane and *b* axis, respectively, with the CDW1 and CDW2 phase transitions indicated by vertical lines. (d) *ρ*(*T*) of NdSeTe_2_ under pressure for *I* || *ac* from 0.4 to 300 K. (e) Low‐temperature part of (d), highlighting the evolution of the superconducting transition with pressure. Insets in (b)–(d): schematic illustrations of the electrical‐contact configurations. Curves in (d) and (e) are vertically offset for clarity.

In this work, we focus on NdSeTe_2_, whose lattice structure is shown schematically in Figure 2a. As in many prototypical CDW systems [1, 2, 3, 4, 5, 6, 7, 8, 9, 10], our electrical transport data reveal a superconducting dome centered near the suppression of CDW order. Surprisingly, however, high‐pressure, low temperature Raman spectroscopy uncovers a crossover from long‐range order to short‐range CDW correlations that persists throughout the superconducting dome (Figure 1b). This observation indicates that the interplay between CDW order and superconductivity is more subtle than commonly assumed, challenging the prevailing CDW‐superconductivity phase diagram.

## 2 Results and Discussion

Figure 2b,c shows the temperature dependence of electrical resistivity *ρ*(*T*) of NdSeTe_2_ at ambient pressure for current applied along the basal plane (*I* || *ac*) and the *b* axis (*I* || *b*), respectively. For *I* || *ac*, *ρ*(*T*) exhibits overall metallic‐like behavior with a hump‐like anomaly at *T*_CDW1_∼260 K. Such a feature is characteristic of the RTe_3_ material systems, where the resistivity anomaly signals the onset of CDW order [20, 24, 25, 26, 27]. In addition, a distinct resistivity anomaly appears at *T*_CDW2_∼215 K in the *ρ*(*T*) curve for *I* || *b*, indicative of a second CDW transition, as confirmed by our Raman measurements below. Double CDW transitions are commonly observed in the heavy rare‐earth members of RTe_3_ with R = Dy‐Tm [20, 24, 25, 26, 27], corresponding to long‐range incommensurate unidirectional and bidirectional CDW orders below *T*_CDW1_ and *T*_CDW2_, respectively.

Figure 2d shows the *ρ*(*T*) curves for *I* || *ac* measured in a DAC with pressures up to 10.0 GPa and temperatures down to 400 mK. Upon compression, *T*_CDW1_ shifts to lower temperatures and the amplitude of the hump‐like resistivity anomaly is reduced progressively; at 3.4 GPa and above, the CDW‐related resistivity anomaly is no longer visible, indicating a gradual suppression and eventual disappearance of CDW order. Concurrently, a small resistivity drop emerges at low temperatures with an onset *T*_C_∼0.7 K at 0.9 GPa and a zero‐resistance state appears at 2.1 GPa, signaling pressure‐induced emergence of superconductivity in NdSeTe_2_. The resistivity drop is suppressed gradually by the application of external fields (Figure S1), further confirming its superconducting nature. *T*_C_ reaches a maximum of ∼1.8 K at *P*_C_ = 2.6 GPa and then starts to decrease with further increasing pressure, forming a dome‐shaped superconducting phase diagram. To track the evolution of the CDW2 order, we measured *ρ*(*T*) for *I* || *b* in a piston cylinder cell at various pressures to 1.8 GPa over the temperature range of 5–300 K (Figure S2). The CDW2 transition is found to be highly pressure‐fragile and is invisible at pressures just under 0.3 GPa. The evolution of the CDW order and superconductivity in compressed NdSeTe_2_ is reproduced in an independent run (Figure S3), demonstrating the robustness of our results. The possibility that the observed superconductivity originates from elemental Te impurities can be excluded, as discussed in detail in Note S1 and Figure S4.

**FIGURE 3:**
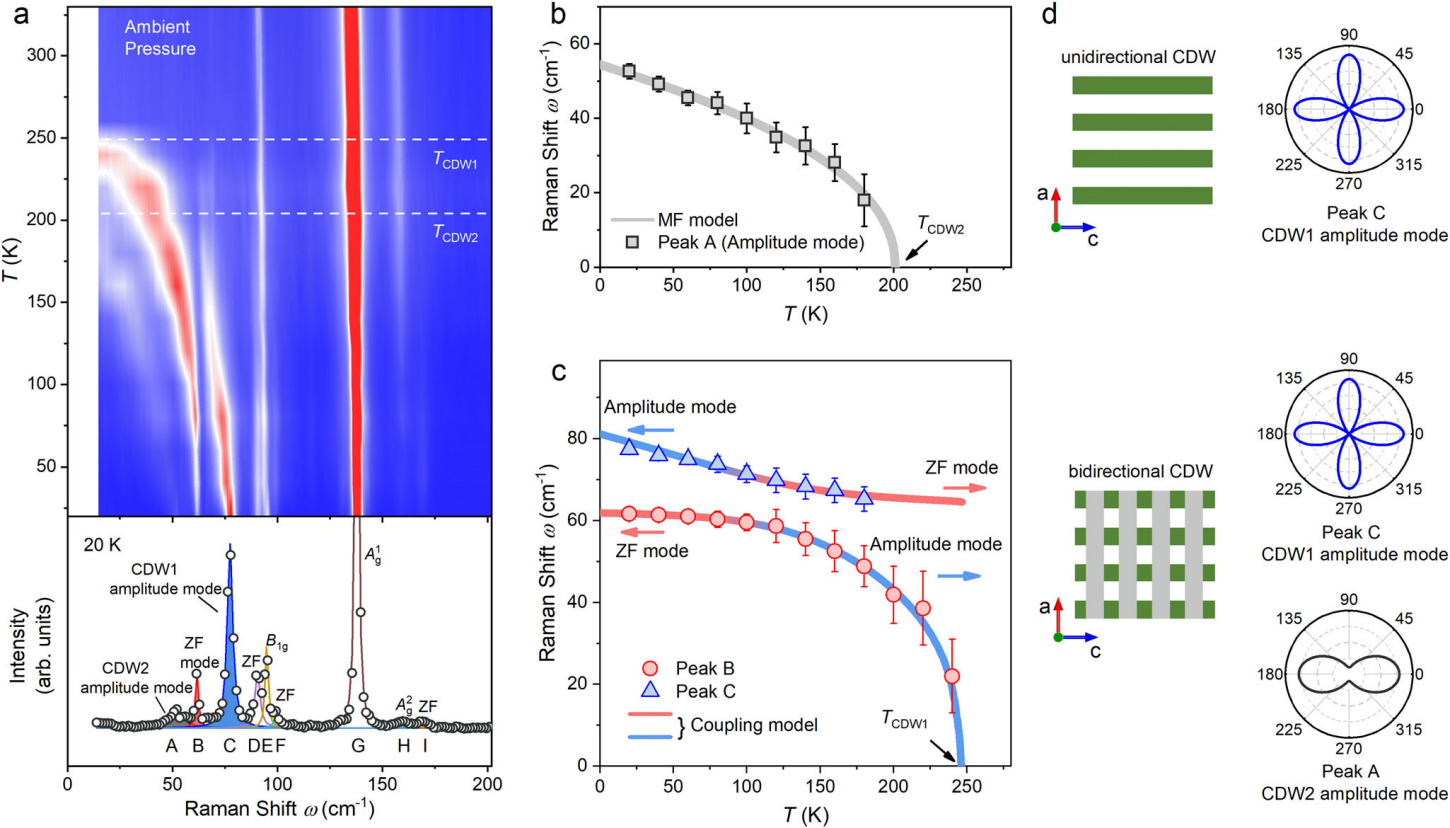
Temperature‐dependent Raman response of NdSeTe_2_ at ambient pressure. (a) Raman spectra of NdSeTe_2_ from 20 to 330 K at ambient pressure. For the sake of the assignment, the 20 K spectrum is plotted separately at the lower panel of (a). (b) Temperature dependence of the Raman shift *ω* of mode A, with the solid line representing the mean‐field (MF) fit. (c) Temperature dependence of the Raman shifts of modes B and C, with solid curves representing fits using the coupling model. (d) Schematic illustration of the distinct angular symmetries of the Raman amplitude‐mode intensities associated with CDW1 and CDW2, corresponding to the incommensurate unidirectional and bidirectional CDW orders that emerge below *T*_CDW1_ and *T*_CDW2_, respectively.

Figure 3a shows the temperature‐dependent Raman spectra of NdSeTe_2_ from 20 to 330 K at ambient pressure. The overall spectral features are consistent with the vibrational characteristics reported for the RTe_3_ family [28, 29, 30, 31, 32]. At 330 K, above *T*_CDW1_, one strong peak (labelled as G) and two weaker peaks (labelled as E and H) are observed, corresponding to Raman‐active phonon modes *A*_g_^1^, *B*_g_^1^, and *A*_g_^2^, respectively. Upon cooling to low temperature (e.g., 20 K, lower panel of Figure 3a), in addition to the three phonon modes, six CDW‐related modes emerge progressively. By definition, the CDW amplitude mode, also referred to as the Higgs mode [33, 34], is a soft phonon associated with the symmetry breaking of the CDW state and reflects amplitude fluctuations of the CDW order parameter, serving as a fingerprint of CDW order [35, 36, 37]. In contrast, the zone‐folded (ZF) modes correspond to normal phonons folded to the Brillouin‐zone center by the CDW superlattice. Accordingly, peaks A and C are amplitude modes, while peaks B, D, F, and I correspond to ZF phonon modes.

Figure 3b,c presents the temperature evolution of the CDW‐related lower‐frequency excitations, that is, modes A, B, and C. Mode A is further identified as the amplitude mode associated with CDW2 and follows mean‐field (MF) behavior of $\omega_{\mathrm{amplitude}}=\omega_{0}{\left(1-\frac{T}{T_{\mathrm{CDW}}}\right)}^{\beta }$, where *ω*_0_ is the Raman shift of CDW amplitude mode at the zero‐temperature limit, and the parameter *β* is a critical exponent. Fitting this temperature dependence in Figure 3b yields *T*_CDW2_ = 202(3) K, slightly lower than the value (215 K) extracted from electrical transport. This discrepancy likely arises from the different uncertainties and criteria used to identify the CDW transition temperature in the two measurements. The behavior of peaks B and C reveals the anticrossing interaction of coupled phonon and amplitude modes, commonly observed in the RTe_3_ family [29, 30, 31, 32, 33, 34]. At low temperature (Figure 3c), the higher‐frequency peak C has an amplitude nature, whereas the lower‐frequency peak B is phononic. Near 100 K, their intensities become comparable, and their characters gradually interchange. According to the coupling model established in earlier Raman studies [31, 32], we obtain the parameter *T*_CDW1_ = 249(5) K, again slightly lower than the value (260 K) obtained from electrical transport.

The incommensurate unidirectional and bidirectional CDW orders that emerge below *T*_CDW1_ and *T*_CDW2_, respectively, exhibit distinct angular symmetries in their corresponding Raman amplitude modes, as schematically illustrated in Figure 3d. Specifically, the CDW1 amplitude mode displays a fourfold angular dependence, while the CDW2 amplitude mode exhibits twofold symmetry. According to a recent study [34] of ErTe_3_ and HoTe_3_, these distinct angular responses may suggest a conventional, scalar character for CDW1 and a lower‐symmetry, possibly unconventional ferroaxial character for CDW2. Nevertheless, further studies are required to establish this interpretation unambiguously. A detailed discussion of the crystal symmetry, Raman selection rules, experimental geometry, polarization configuration, definition of the rotation angle, and atomic displacement patterns of the principal phonon modes is provided in Note S2, Figures S5 and S6.

**FIGURE 4:**
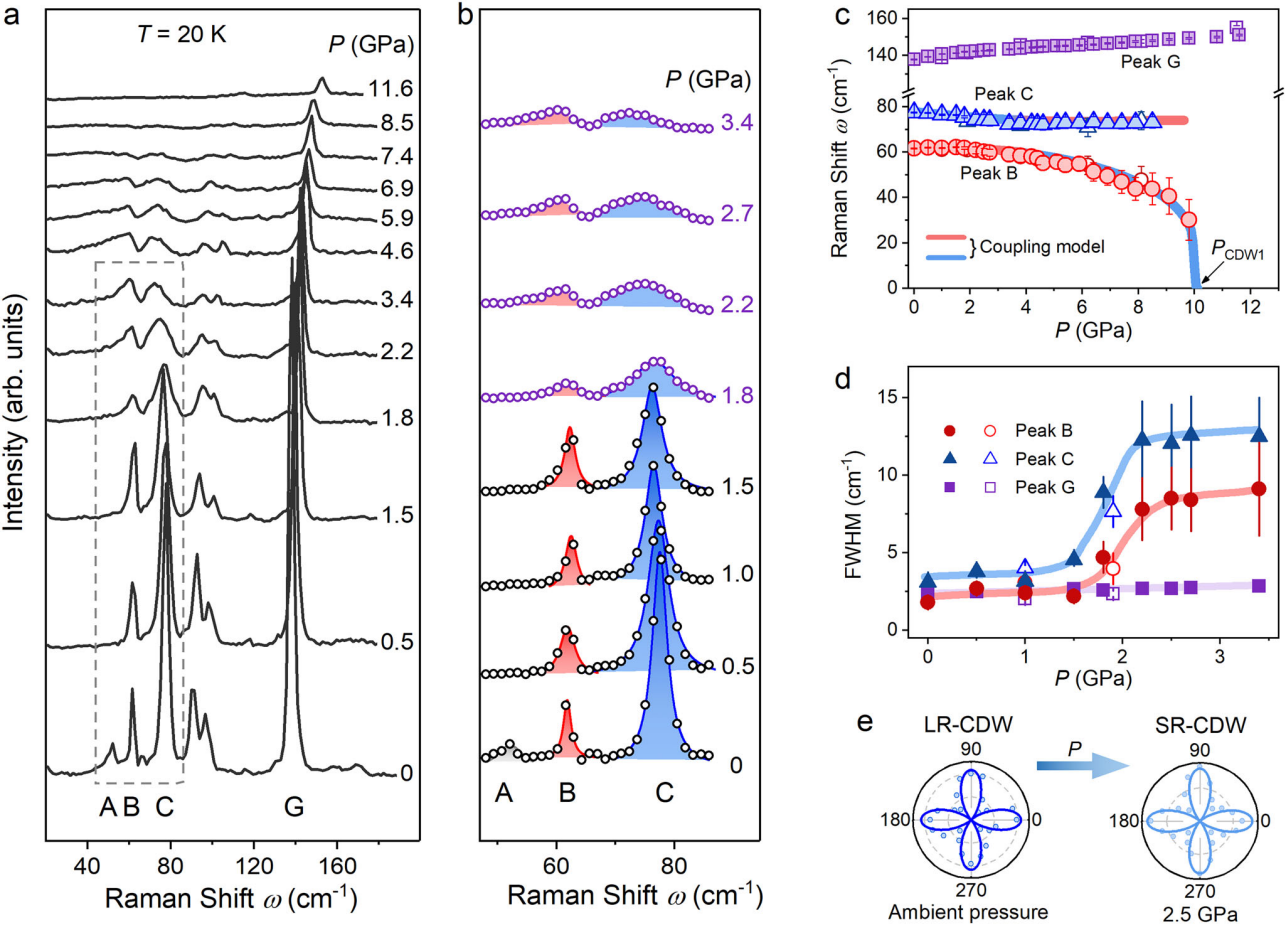
High‐pressure Raman spectra at 20 K. (a) Raman spectra of NdSeTe_2_ at 20 K under selected pressures. The CDW‐related modes A, B, and C are fitted with Lorentzian profiles to extract peak positions and full widths at half maximum (FWHM). (b) Enlarged view of the Raman spectra near 70 cm^−1^, highlighting the abrupt broadening of the CDW1‐related modes around 2 GPa. (c) Pressure evolution of the Raman shifts of peaks B, C, and G, with solid curves corresponding to fits using the coupling model. (d) Pressure dependence of the FWHM of peaks B, C, and G, showing a pronounced anomaly near 2 GPa that signals the crossover from long‐range to short‐range CDW order. Solid and empty symbols denote data from different runs. (e) Polar plots of the polarized intensity of mode C at ambient pressure and 2.5 GPa in the parallel polarization configuration.

After identifying all Raman‐active modes, we carried out temperature‐dependent Raman measurements under pressure to trace the evolution of the CDW states. As shown in Figure 4a,b, the most notable change between 0 and 0.5 GPa at 20 K is the disappearance of the CDW2 amplitude mode (Peak A). Together with electrical transport data obtained in the piston cylinder cell (Figure S2), this indicates a rapid collapse of the CDW2 order upon initial compression (below 0.5 GPa) from both the electronic and lattice degrees of freedom.

With further increasing pressure, the CDW1 amplitude mode (Peak C) evolves as expected for a pressure‐suppressed CDW state (Figure 1a), softening toward zero energy (Figure 4a). Surprisingly, however, the CDW1‐related modes (peaks B and C) persist substantially above the nominal *P*_C_ (Figure 4b). An anticrossing between the amplitude mode (peak C) and ZF phonon mode (Peak B) occurs near 4 GPa, mirroring the behavior observed upon warming. A fit in terms of the above‐mentioned coupling model but with respect to pressure (Figure 4c) yields the genuine critical pressure *P*_CDW_ = 10.1(2) GPa. Meanwhile, the full width at half maximum (FWHM) of peaks B and C increases by nearly a factor of three on crossing *P*_C_ (Figure 4d); in stark contrast, the Raman shift and FWHM of the phonon mode G evolve smoothly with pressure and show no evident anomalies (Figure 4c,d). This indicates substantial additional broadening of peaks B and C across *P*_C_, despite the reduced accuracy due to the presence of an anticrossing effect. Full Raman spectra under different pressure and temperature conditions are given in Figure S7 (an independent run), which reproduce all the above results.

One may be concerned whether this broadening could result from pressure inhomogeneity rather than an intrinsic change in the CDW state. To address this issue, we performed an independent Raman measurement using Daphne 7373 oil, which provides a more hydrostatic pressure environment than NaCl used in the above measurements. The pronounced broadening of the CDW‐related modes near *P*_C_ was reproduced in this measurement, as shown in Figure S8 of the Supporting Information. By contrast, the ordinary Raman‐active phonon mode G exhibits only a weak and smooth pressure dependence of its linewidth. This mode‐selective response indicates that the abrupt broadening is primarily associated with the CDW sector rather than with a general deterioration of sample quality or pressure homogeneity.

Together with the disappearance of a clear CDW anomaly in resistivity, the persistence of broadened CDW‐related Raman modes above *P*_C_ suggests a crossover from long‐range CDW order to short‐range CDW correlations. Across *P*_C_, the mode frequencies and fourfold angular dependence change only weakly, indicating that the local CDW energy scale and orientational symmetry remain largely preserved. In contrast, the pronounced linewidth broadening points to a substantial reduction in CDW coherence and/or collective‐mode lifetime. Although Raman spectroscopy does not directly determine the spatial correlation length, the combined transport and Raman results are consistent with the persistence of local CDW correlations after the loss of macroscopic long‐range coherence.

Similar behavior has been reported in other CDW systems. In 1*T*‐NbSeTe, the transport signature associated with long‐range CDW order disappears while local CDW correlations remain detectable by spectroscopic and microscopic probes [38, 39]. Likewise, across the commensurate‐to‐nearly‐commensurate CDW crossover in 1*T*‐TaS_2_, the CDW‐related Raman modes broaden markedly while their frequencies change only modestly, indicating that the loss of long‐range coherence affects the collective‐mode linewidth more strongly than the local CDW energy scale [40]. In addition, a long‐to‐short range CDW crossover has also been reported in Pd‐intercalated ErTe_3_ [41] and Cu‐intercalated TiSe_2_ [13, 14, 15], where quenched disorder introduced by intercalation plays a key role.

We also performed angle‐resolved Raman measurements in parallel‐polarization geometry at 20 K to track the symmetry of the CDW orders across *P*_C_ (Figure S6). At ambient pressure, the amplitude modes associated with the unidirectional CDW1 and bidirectional CDW2 orders in NdSeTe_2_ exhibit distinct rotational patterns: CDW1 shows fourfold symmetry, while CDW2 displays twofold symmetry (Figure 3d and Figure S6). A comparison between ambient‐pressure spectra and those at 2.5 GPa demonstrates that the fourfold symmetry of the CDW1 amplitude mode is essentially preserved (Figure 4e), indicating no additional symmetry change upon crossing *P*_C_. Note that the rotational symmetries of the CDW amplitude modes in NdSeTe_2_ are reversed from those in heavy RTe_3_ compounds exhibiting two CDW transitions (e.g., ErTe_3_ [34]), where the high‐temperature CDW1 amplitude mode is twofold, and the low‐temperature CDW2 one is fourfold. This contrast suggests that in NdSeTe_2_ the CDW1 wave vector lies along the *a* axis (unidirectional), whereas the CDW2 order involves a bidirectional modulation along the *c* axis. This is further supported by extrapolating the doping dependence of *T*_CDW1_ and *T*_CDW2_ in the series of RTe_3_ compounds (Figure S9), which reveals a crossing of the two transition temperatures at nearly the same in‐plane lattice parameter of ambient‐pressure NdSeTe_2_ (the average lattice constant *ã* ≈ 4.26 Å).

**FIGURE 5:**
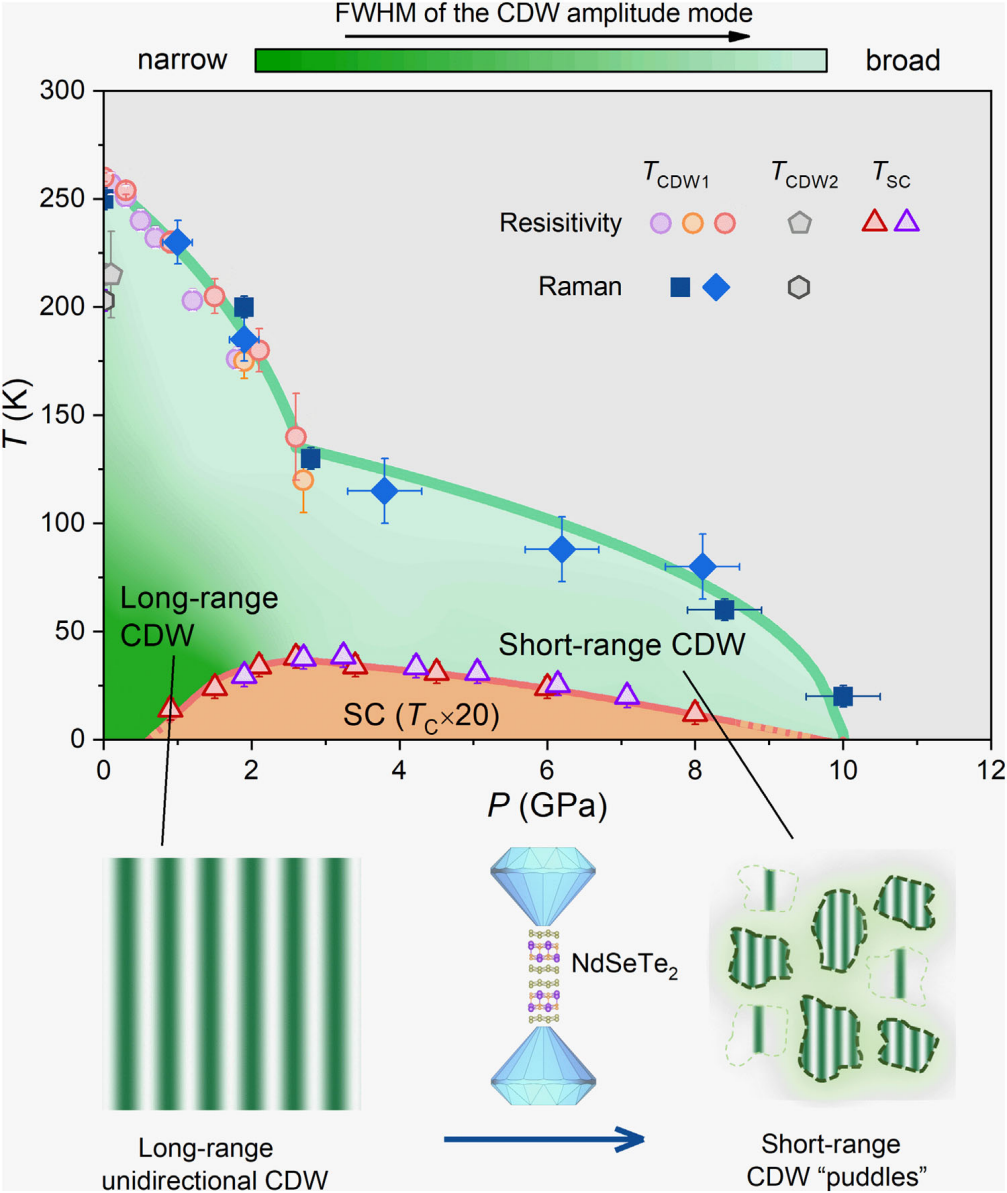
Temperature‐pressure phase diagram showing the CDW transition temperatures *T*_CDW1_ and *T*_CDW2_, together with the superconducting transition temperature *T*_C_. The color scale within the CDW region represents the Raman FWHM of the CDW amplitude mode. Schematic illustrations below the phase diagram depict the proposed evolution of the CDW state under compression: at low pressure, the CDW forms long‐range unidirectional order (green stripes), whereas at high pressure, short‐range correlations persist with a puddle‐like spatial texture. SC denotes superconductivity.

Based on the above experimental measurements, we construct the *T–P* phase diagram of NdSeTe_2_, which summarizes the evolution and interplay of CDW order and superconductivity (Figure 5). The symbols used in this figure are defined in Figure S10. Below *P*_C_, the *T*_CDW1_ values extracted from Raman spectroscopy agree well with those from electrical transport, both showing a monotonic decrease under compression. Along with suppression of CDW1 order, superconductivity emerges, with *T*_C_ increasing and reaching its maximum at *P*_C_. Our high‐pressure, low‐temperature Raman measurements reveal that the pristine CDW does not vanish across *P*_C_; instead, a crossover from long‐range CDW order to short‐range CDW correlations occurs. Note that the precise value of *P*_C_ differs slightly depending on whether it is inferred from transport or Raman measurements. Here, *P*_C_ is used as a characteristic pressure to emphasize that optimal superconductivity emerges concomitantly with the loss of long‐range CDW coherence, rather than to imply that the crossover occurs sharply at a single pressure. Remarkably, the short‐range CDW correlations persist up to ∼10 GPa and coexist with superconductivity over the entire dome.

The pressure dependence of *T*_CDW_ changes across *P*_C_. Above *P*_C_, the Raman‐derived *T*_CDW_ should be interpreted as a characteristic temperature below which short‐range CDW correlations become detectable, rather than as a sharp thermodynamic transition temperature. The apparent change in slope near *P*_C_ therefore reflects a crossover from long‐range CDW order to short‐range CDW correlations, which possess distinct correlation lengths and pressure sensitivities. An analogous behavior has been reported in pressurized 1*T*‐TaS_2_, where the transition temperature of the commensurate CDW decreases rapidly with pressure, whereas the nearly commensurate CDW regime evolves much more gradually [42].

Our in situ high‐pressure synchrotron XRD measurements at room temperature reveal no structural transition or anomaly with pressures up to 32.4 GPa (Note S1 and Figure S11); likewise, the pressure evolution of the position and FWHM of the Raman‐active phonon mode G at 20 K shows no evident discontinuity across *P*_C_ (Figure 4c,d). These results imply that the pronounced FWHM anomaly of the CDW amplitude mode near *P*_C_ is electronic in origin rather than driven by a structural transition. In CDW systems such as Pd_x_ErTe_3_ [41, 43] and Cu_x_TiSe_2_ [13, 14, 15], intercalation introduces disorder that frustrates long‐range CDW order and pins short‐range correlations. In NdSeTe_2_, the in‐plane parameters at ambient pressure are nearly equal (*c* ≅ *a*), distinguishable from its counterparts—heavy RTe_3_ compounds exhibiting two CDW transitions (Figure S9). Previous studies have shown that when *c* ≅ *a*, the unidirectional CDWs along the *a* and *c* axes are energetically comparable and can be reversibly switched by uniaxial stress, as demonstrated in some heavy RTe_3_ compounds [24, 44, 45] and in Pd_x_ErTe_3_ [46]. This suggests that the two competing unidirectional CDWs should have similar ground‐state energies. We therefore argue that their competition in compressed NdSeTe_2_ plays a role analogous to quenched disorder introduced by intercalation or strain, frustrating the system and driving a fragmentation of the CDW from long‐range coherence to a short‐range state. A theoretical study [47] has shown that in quasi‐one‐dimensional CDW systems, enhanced coupling between CDW stripes amplifies dynamical fluctuations and reduces the CDW stiffness; these long‐wavelength fluctuations can destroy long‐range translational order while preserving long‐range orientational order. This expectation is consistent with our observation that the rotational symmetry of the CDW amplitude mode remains unchanged (Figure 4e). The superconducting coherence length at the zero‐temperature limit *ξ*_GL_(0) is estimated to span approximately 135–180 nm across the full superconducting dome. Taken together, these results imply that the short‐range CDW persisting at high pressure retains a unidirectional *a*‐axis wave vector and likely manifests as similar scale (i.e., nanoscale) CDW “puddles”, as schematically shown in the lower panel of Figure 5, reminiscent of those observed in cuprates [48, 49, 50] and Cu_x_TiSe_2_ [13, 14, 15].

As noted in the Introduction, many prototypical CDW materials appear to show a “universal” trend in which optimal superconductivity emerges as CDW order is suppressed, yielding a dome‐shaped superconducting phase diagram [1, 2, 3, 4, 5, 6, 7, 8, 9, 10]. However, this picture is based almost entirely on electrical transport. Our observation of a non‐universal behavior in compressed NdSeTe_2_, that is, the persistence of CDW order across the entire superconducting dome, demonstrates that transport alone can provide an incomplete or even misleading view. Across a wide range of systems, including cuprates [48, 51, 52], transition‐metal dichalcogenides [12, 13, 14, 15], and members of the RTe_3_ family [41, 43], superconductivity often emerges in proximity to short‐range or fluctuating CDW correlations. In these material systems, CDW inhomogeneity, either arising from quenched disorder or from coexisting commensurate and incommensurate CDW orders, is believed to play a central role. A similar scenario may apply to compressed NdSeTe_2_ where optimal superconductivity with the highest *T*_C_ appears coincidentally as short‐range CDW order becomes prominent near *P*_C_, a regime in which CDW inhomogeneity is likely maximal. The resulting inhomogeneous CDW state coexists with superconductivity in an electronically phase‐separated manner. Notably, in Cu‐intercalated TiSe_2_ [13, 14, 15], short‐range charge order was also found to persist across the superconducting dome, but only through the combination of transport and structure‐sensitive probes. Nevertheless, the coexistence regime is confined to a relatively narrow portion of the dome‐shaped phase diagram, rendering it a precursor observation. Taken together, the convergence of evidence across these disparate systems emphasizes the essential need to track CDW evolution simultaneously in the electronic and lattice channels. More broadly, it may represent a canonical *intrinsic* feature of CDW‐superconductivity interplay in low‐dimensional quantum systems, contrary to the current widely‐accepted paradigm.

## 3 Conclusion

To summarize, we have established the complete *P–T* phase diagram of NdSeTe_2_ through combined electrical transport, Raman spectroscopy, and synchrotron XRD measurements. While resistivity alone suggests that a superconducting dome forms as the long‐range CDW order is suppressed at a critical pressure *P*_C_, our low‐temperature Raman data reveal that short‐range CDW correlations persist well beyond this point, evidenced by pronounced broadening of the CDW‐related amplitude mode. This short‐range CDW survives even up to ∼10 GPa, spanning the full superconducting dome. The coincidence of optimal superconductivity with the onset of short‐range CDW correlations indicates that superconductivity is promoted by the inhomogeneous CDW landscape. Such an unusual relationship between CDW order and superconductivity in compressed NdSeTe_2_, that is, a persistent CDW encompassing the entire superconducting dome, calls for a reconsideration of their coupling in shaping the phase diagram. It may even suggest that such behavior is potentially generic among CDW materials exhibiting a full superconducting dome once complementary probes are employed.

## 4 Experimental Section

### 4.1 Sample Synthesis

Single crystals of NdSeTe_2_ were synthesized using the LiCl/RbCl flux method [53]. Stoichiometric amounts of neodymium lump, selenium powder, and tellurium powder, along with a tenfold excess of dried LiCl/RbCl, were sealed in an evacuated quartz tube. The mixture was heated to 700°C over 7 h, held for 72 h, then slowly cooled to 300°C over 300 h before a natural cooldown to room temperature. Plate‐like specimens with a golden surface were mechanically cleaved from the obtained single crystals by Scotch tape. All preparation steps were carried out in an argon‐filled glove box.

### 4.2 Electrical Transport Measurements

Standard four‐probe configurations were used for both ambient‐ and high‐pressure transport measurements with *I* // *ac*. For measurements with *I* // *b*, the current electrodes were attached to opposite faces of the specimen at one side, while the voltage electrodes were attached to opposite faces at the other side. High‐pressure electrical transport experiments were performed using a nonmagnetic Be‐Cu alloy diamond anvil cell (DAC) with NaCl powder as the pressure‐transmitting medium. Sub‐kelvin temperatures down to 400 mK were achieved in a 9‐T C‐MAG cryostat (CRYOMAGNETICS) equipped with an ^3^He insert. Ruby fluorescence was used to determine pressure at room temperature for transport.

### 4.3 Synchrotron XRD Measurements

High‐pressure angle‐dispersive synchrotron X‐ray diffraction (XRD) was conducted in a Mao‐Bell‐type DAC at beamline BL15U1 of the Shanghai Synchrotron Radiation Facility (SSRF), using a monochromatic X‐ray beam with wavelength 0.6199 Å and Daphne 7373 as the pressure‐transmitting medium. Ruby fluorescence was used to determine pressure at room temperature for XRD.

### 4.4 Raman Measurements

Ambient‐ and high‐pressure Raman measurements were performed with Renishaw instruments in a backscattering geometry using a 532‐nm laser. The laser powder was kept at 2.5 mW. Laser heating effect was assessed from the anti‐Stokes/Stokes intensity ratio. For variable‐temperature high‐pressure Raman experiments, a freshly cleaved crystal was loaded into a Be‐Cu DAC with NaCl or Daphne 7373 as the pressure medium, and cooled in a JANIS‐ST500 cryostat. Pressure at low temperature was adjusted by the He gas membrane method. Ruby fluorescence was used to determine pressure at low temperature for Raman measurements.

## Author Contributions

**Shuyang Wang**: investigation, methodology, data curation, writing – original draft, writing – review and editing, formal analysis, visualization. **Chao An**: methodology, visualization. **Jiaqi Chen**: methodology. **Lili Zhang**: methodology. **Yonghui Zhou**: methodology. **Xuliang Chen**: conceptualization, funding acquisition, writing – original draft, writing – review and editing, data curation, supervision, project administration, formal analysis. **Zhaorong Yang**: supervision, project administration, writing – review and editing, funding acquisition, formal analysis.

## Conflicts of Interest

The authors declare no conflicts of interest.

## Supporting information




**Supporting File**: advs77597‐sup‐0001‐SuppMat.pdf.

## Data Availability

The data that support the findings of this study are available from the corresponding author upon reasonable request.
