# Supplementary material for: Pressure‐Tuned Superconducting Dome and Persistent Charge‐Density‐Wave Order in NdSeTe2
Source: Adv Sci (Weinh). 2026 Sep 3:e77597. Online ahead of print. doi: 10.1002/advs.77597 (PMC13542405; doi:10.1002/advs.77597)
Supplement: Supplementary file 1 [file ADVS-9999-e77597-s001.pdf]

Supporting Information

Pressure-Tuned Superconducting Dome and Persistent Charge-Density-Wave Order in $NdSeTe_2$

Shuyang Wang, Chao An, Jiaqi Chen, Lili Zhang, Yonghui Zhou, Xuliang Chen, and Zhaorong Yang**

This PDF file includes:

Notes S1 to S3
Figures S1 to S11
References 1 to 8

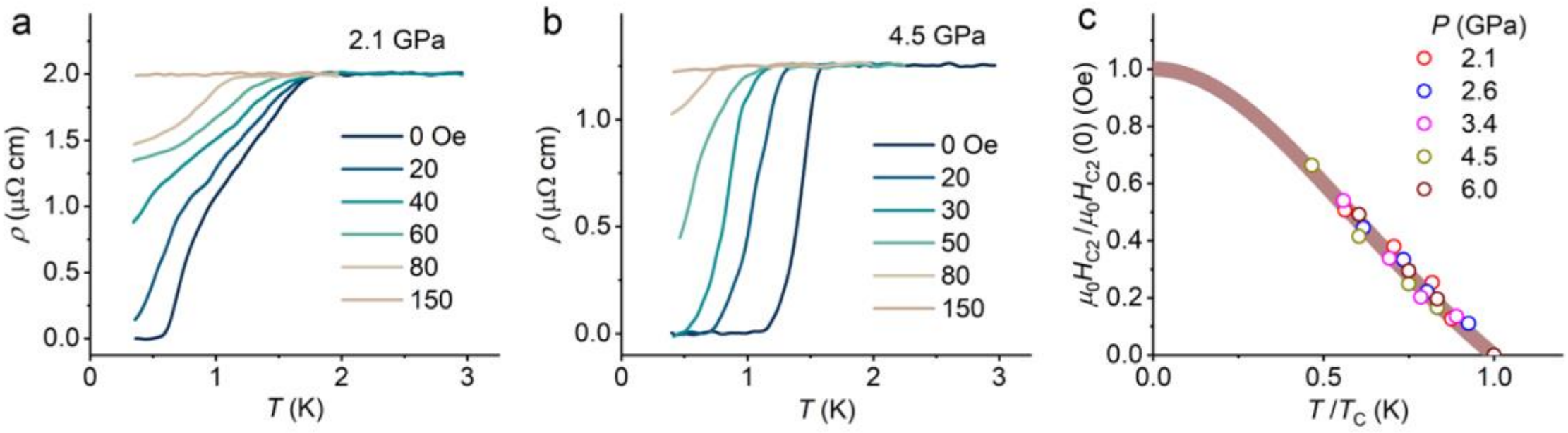

Figure S1. a) and b) Temperature dependence of resistivity $\rho(T)$ at selected pressures of 2.1 and 4.5 GPa, respectively. c) Reduced temperature-field phase diagram for $NdSeTe_2$ at various pressures. Solid lines denote fits using the Ginzburg–Landau model to estimate the upper critical field μ_0H_{c2} at zero temperature.

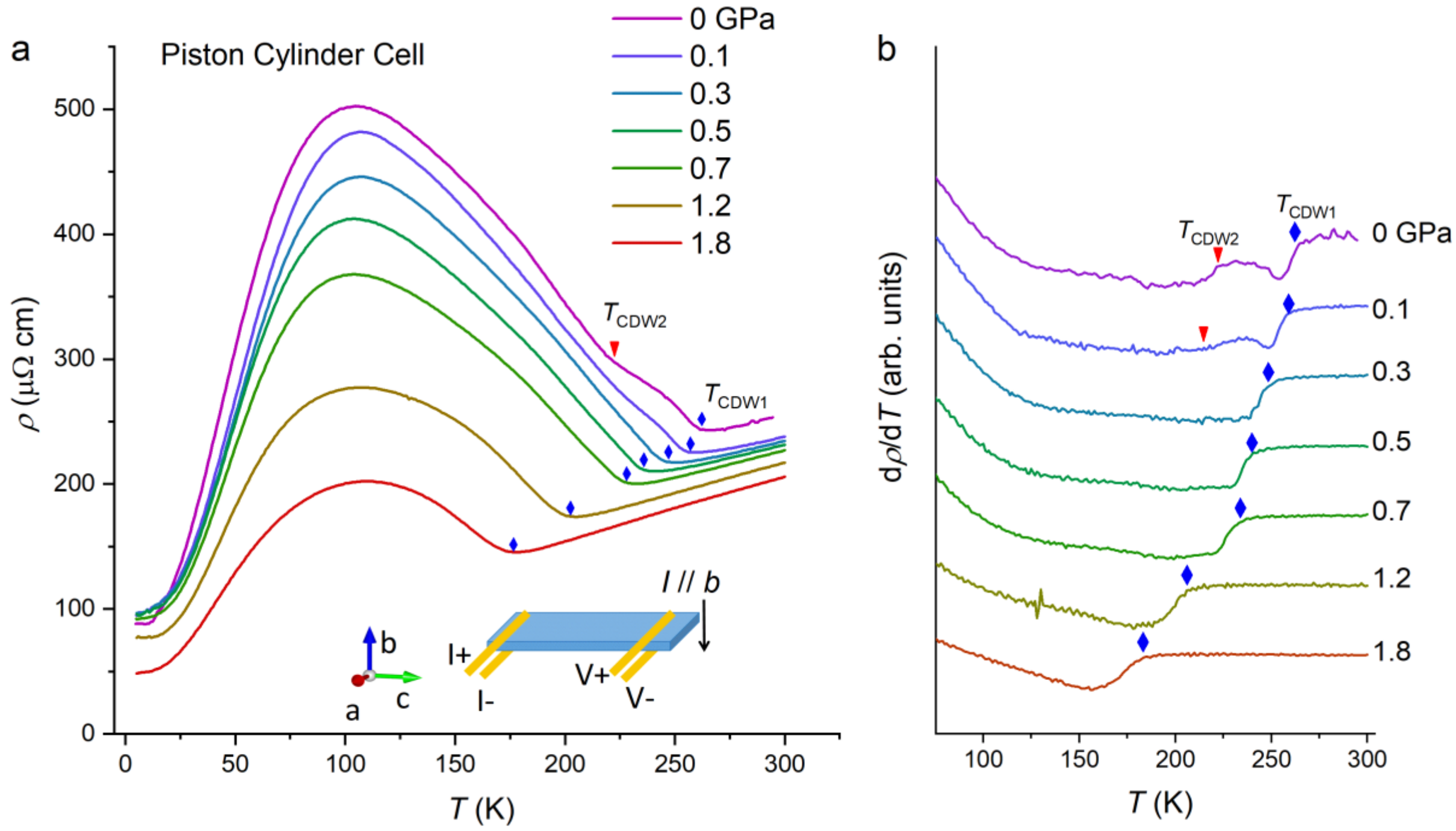

Figure S2. a) Temperature dependent resistivity $\rho(T)$ for $I \parallel b$ under different pressures measured in a piston cylinder cell and b) its first-order derivative dρ/dT. All curves in (b) are offset vertically for clarity.

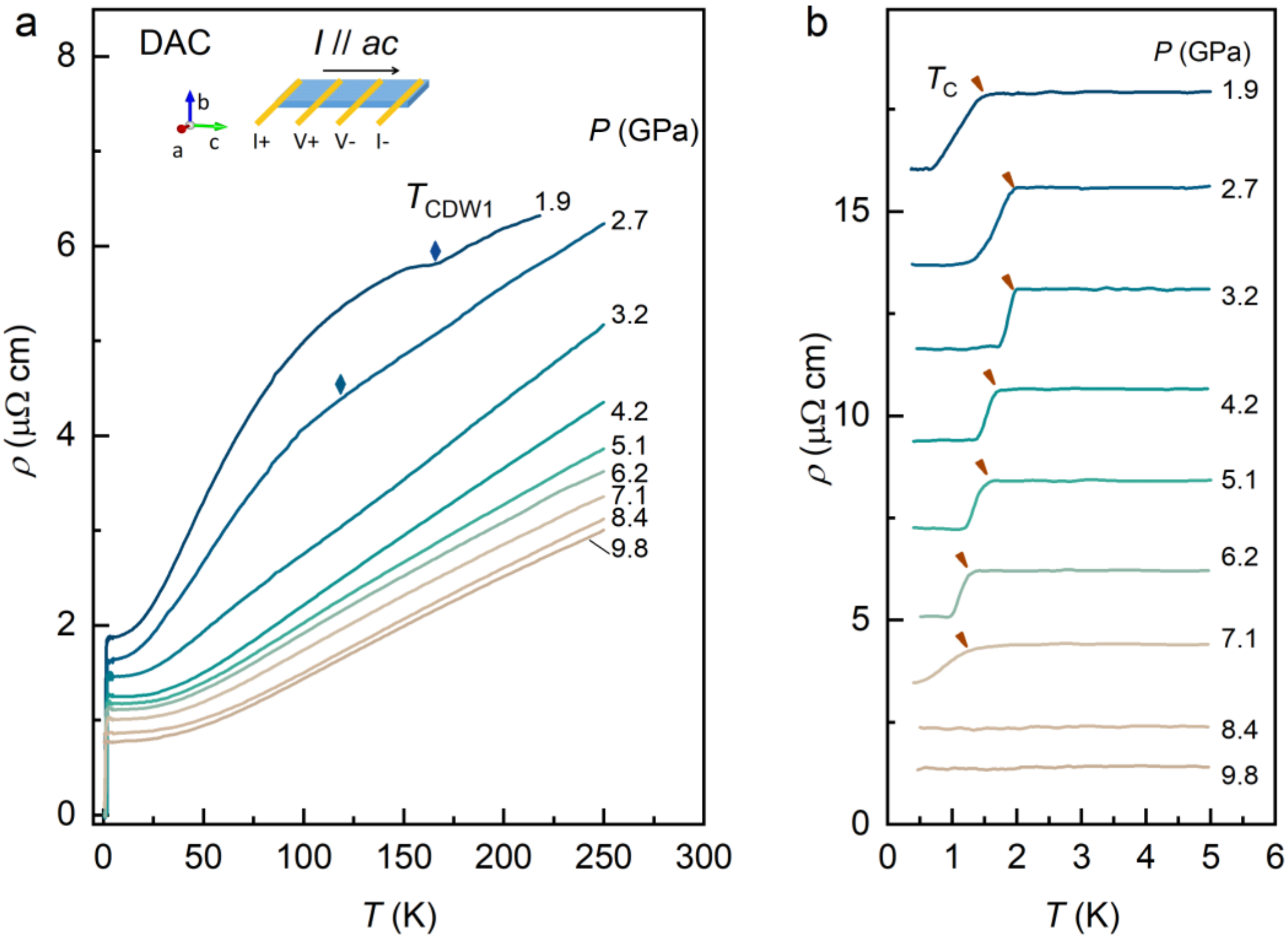

Figure S3. a) Temperature dependence of resistivity $\rho(T)$ for $I \parallel ac$ under different pressures measured in a DAC (an independent run). b) Low-temperature part of (a), focusing on pressure evolution of superconductivity. Curves are shifted vertically for clarity.

Note S1: Excluding the possibility that the observed superconductivity originates from elemental Te impurities

First, $NdSeTe_2$ single crystals were grown using a LiCl/RbCl salt-flux method rather than the conventional Te self-flux method,[1] substantially reducing the likelihood of residual elemental Te in the resulting crystals. Second, we compare the $T_C(P)$ phase diagrams of $NdSeTe_2$ and elemental Te,[2-5] as shown below (Figure S4). The superconducting phase diagrams are clearly distinct: superconductivity emerges near 1 GPa in $NdSeTe_2$, compared with approximately 4 GPa in elemental Te; it disappears near 10 GPa in $NdSeTe_2$, while superconductivity in Te persists beyond 15 GPa; and the maximum T_C values are approximately 2 and 5 K, respectively. The overall $T_C(P)$ dependences also differ substantially.

Furthermore, no diffraction peaks attributable to elemental Te are detected in the ambient-pressure powder X-ray diffraction pattern, and all high-pressure diffraction patterns can be indexed using the *Cmcm* structure of $NdSeTe_2$, without evidence of an additional Te phase. Taken together, the distinct superconducting phase diagram, the salt-flux growth method, and the X-ray diffraction results demonstrate that the observed superconductivity is intrinsic to $NdSeTe_2$, rather than arising from elemental Te inclusions.

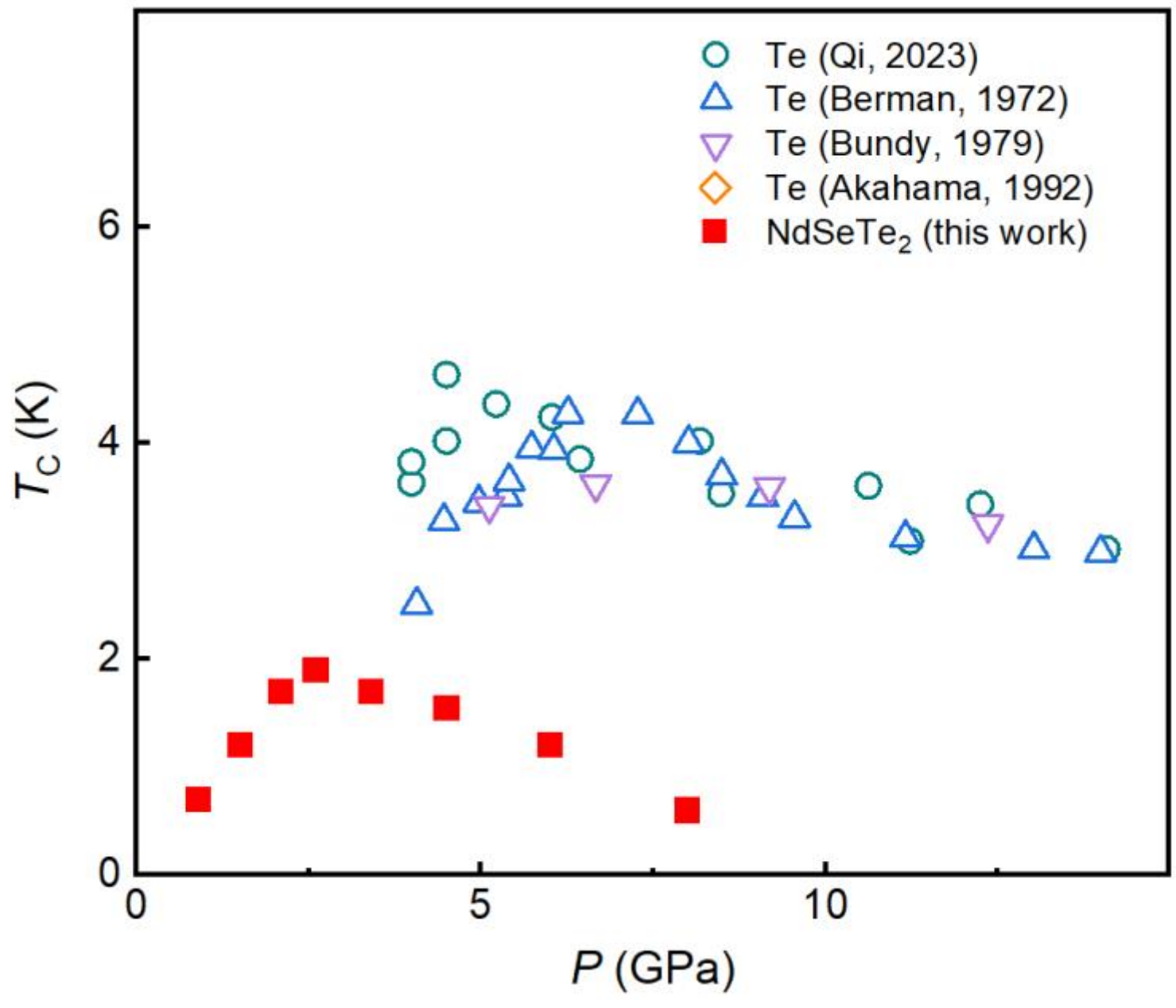

Figure S4. Comparison of the temperature-pressure phase diagrams of $NdSeTe_2$ (solid symbols) and elemental Te (open symbols).

Note S2: Details of the Raman-scatter analysis

At high temperature, undistorted $NdSeTe_2$ crystallizes in the orthorhombic *Cmcm* space group, corresponding to the D_{2h} point group. Factor group analysis yields 12 Raman-active phonons modes [6],

$$\Gamma = 4A_g + 4B_{1g} + 4B_{3g}.$$

Polarized Raman measurements were performed in backscattering geometry from the (010) crystal surface, with the incident and scattered light in the parallel-polarization configuration. Because the crystallographic axes cannot be determined directly once the sample is loaded into the diamond anvil cell, the precise in-plane orientation of the crystal is not independently known. By comparison with previous polarized Raman measurements, the reference polarization direction is inferred to lie approximately along either the [100] or [001] crystallographic axis.

The Raman tensors for the relevant Raman-active modes are

$$R(A_g) = \begin{pmatrix} a & 0 & 0 \\ 0 & b & 0 \\ 0 & 0 & c \end{pmatrix}, R(B_{1g}) = \begin{pmatrix} 0 & d & 0 \\ d & 0 & 0 \\ 0 & 0 & 0 \end{pmatrix}, \text{and } R(B_{3g}) = \begin{pmatrix} 0 & 0 & 0 \\ 0 & 0 & e \\ 0 & e & 0 \end{pmatrix}.$$

For light incident normal to the ac plane in the parallel-polarization configuration, the incident and scattered polarization vectors are

$$\hat{e}_i = \hat{e}_s = (\cos\theta, 0, \sin\theta),$$

where $\hat{e}_i$ and $\hat{e}_s$ denote the polarization unit vectors of the incident and scattered light, respectively, and θ is the angle between the light polarization and an in-plane principal crystallographic axis. The corresponding Raman intensities are

$$I(A_g) = |(a\cos^2(\theta)) + (c\sin^2(\theta))|^2,$$

$$I(B_{1g}) = I(B_{3g}) = 0.$$

Thus, only A_g modes are expected in this scattering geometry. The following schematic (Figure S5) shows the atomic displacement patterns of the main Raman-active A_g modes observed in $NdSeTe_2$. The structure is projected onto the ab plane, with dark and light colors denoting atoms with positive and negative *c* coordinates, respectively. Solid lines indicate the primitive unit cell.

Nevertheless, B_{1g} can be observed because the vibrational response of RTe_3-related compounds is often governed by the approximate tetragonal symmetry of the individual Te-containing layers rather than by the weak orthorhombicity of the three-dimensional crystal [6, 7]. The weakly coupled layered structure can be treated using the diperiodic layer group, for which factor-group analysis predicts four Raman-active modes,

$$\Gamma_{\text{Raman}}{}^{\text{layer}} = 3A_{1g} + B_{1g}.$$

For the approximate D_{4h} symmetry of the Te square net, the Raman tensors for $NdSeTe_2$ are

$$R(A_{1g}) = \begin{pmatrix} a & 0 & 0 \\ 0 & b & 0 \\ 0 & 0 & a \end{pmatrix} \text{ and } R(B_{1g}) = \begin{pmatrix} c & 0 & 0 \\ 0 & 0 & 0 \\ 0 & 0 & -c \end{pmatrix}.$$

For parallel polarization with $\hat{e}_i = \hat{e}_s = (\cos\theta, 0, \sin\theta)$, the Raman intensities are

$$I(A_{1g}) = |(a\cos^2(\theta)) + (a\sin^2(\theta))|^2 = |a|^2,$$

and

$$I(B_{1g}) = |(c\cos^2(\theta)) - (c\sin^2(\theta))|^2 = |c\cos 2(\theta)|^2.$$

Thus, an A_{1g} mode is isotropic in the parallel configuration, while a B_{1g} mode exhibits a fourfold angular dependence. These selection rules agree with the present and previous angle-resolved Raman studies of RTe_3.

Upon entering the CDW state, the symmetry is reduced. A recent study of RTe_3 distinguishes between conventional and unconventional CDW orders [7]. A conventional unidirectional CDW breaks translational symmetry along q_{CDW}, reduces the rotational symmetry from C_4 to C_2, and breaks the diagonal mirror symmetries, while preserving the vertical mirror planes. By contrast, an unconventional ferroaxial CDW additionally breaks the vertical mirror symmetries while preserving inversion and time-reversal symmetries. Our polarized Raman measurements (Figure S6) show that the high-temperature CDW1 amplitude mode exhibits fourfold symmetry, whereas the low-temperature CDW2 amplitude mode exhibits twofold symmetry. Within the symmetry framework established for RTe_3, these contrasting angular responses are consistent with a conventional, scalar character for CDW1 and a lower-symmetry, potentially unconventional character for CDW2. However, because the antisymmetric tensor component cancels in the parallel-polarization channel, crossed- or helicity-resolved Raman measurements would be required to establish unambiguously whether CDW2 is ferroaxial.

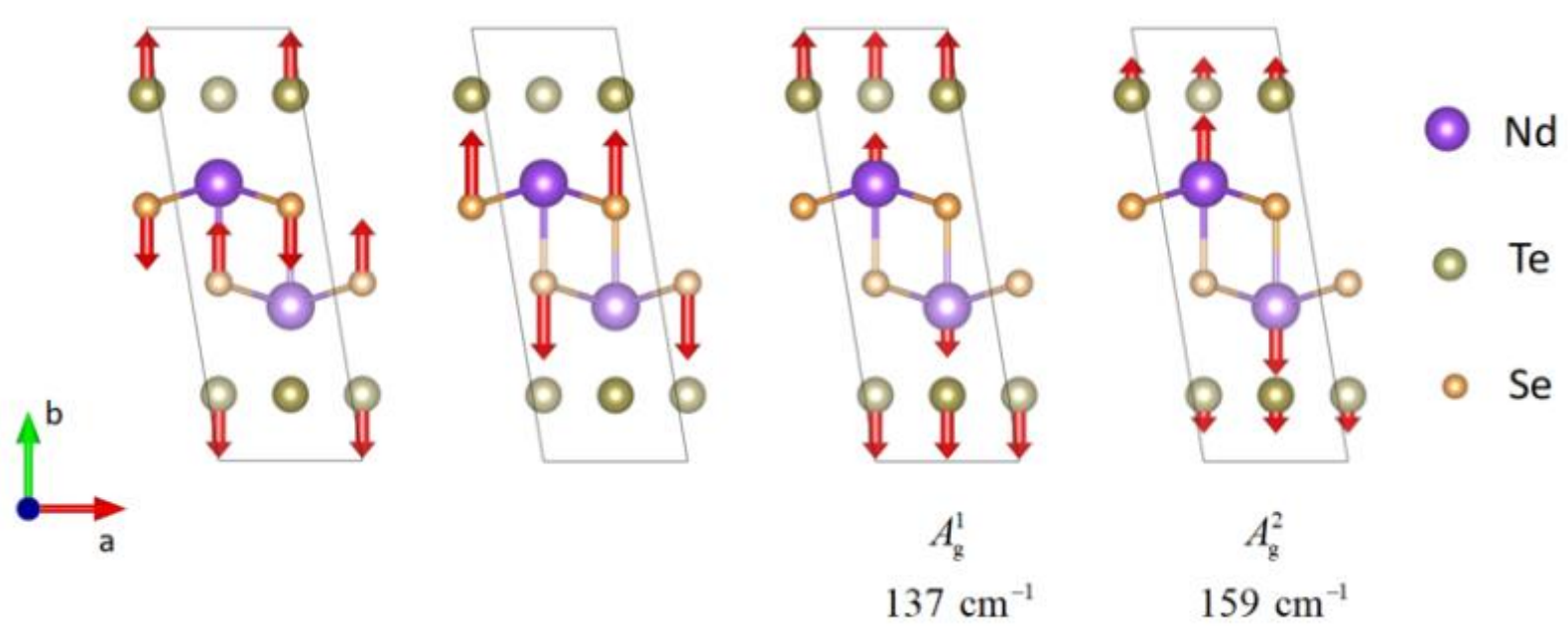

Figure S5. Atomic displacements for the Raman-active A_g vibrational modes of $NdSeTe_2$.

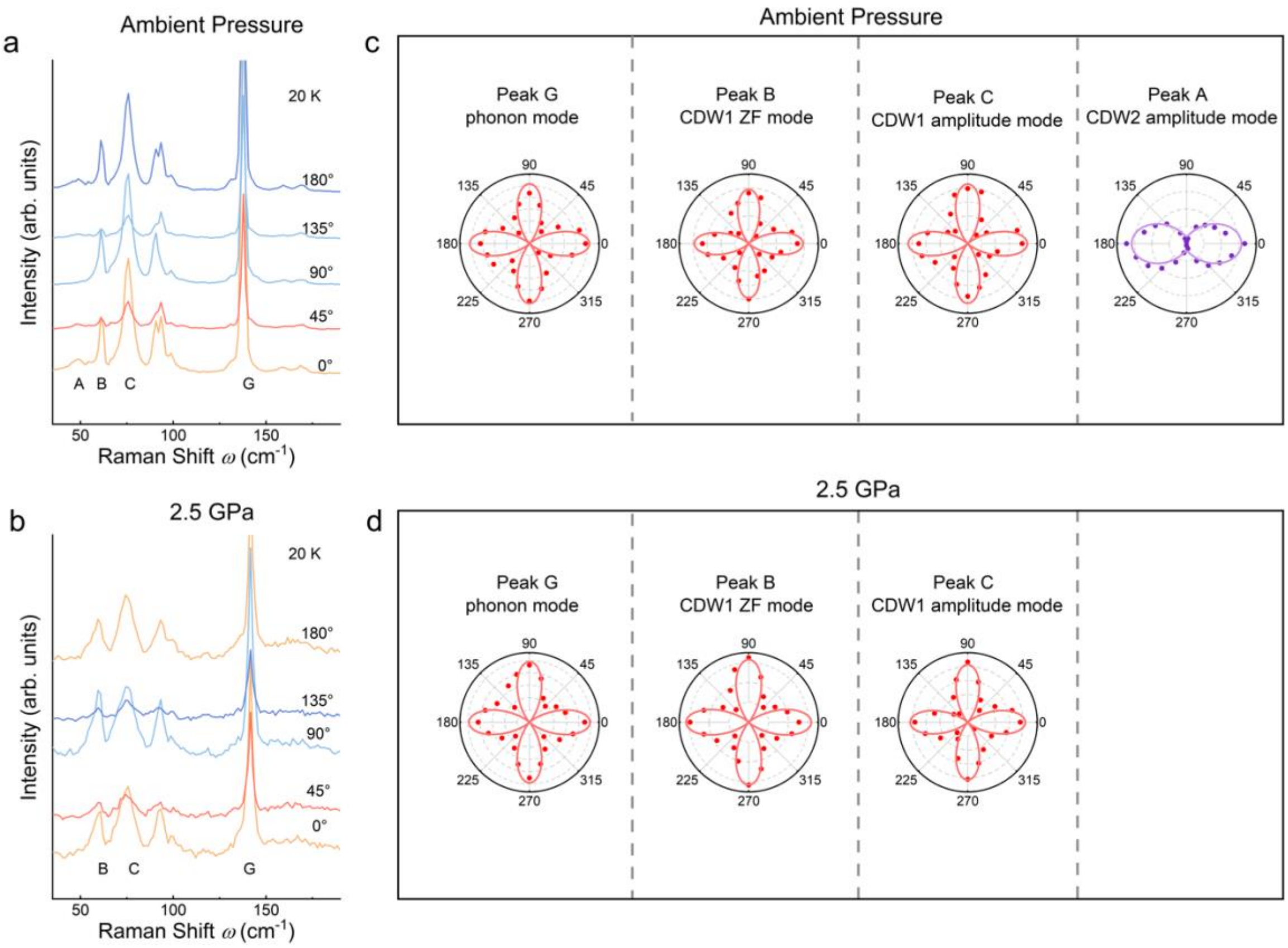

Figure S6. a) and b) Polarization-angle-dependent Raman spectra in the parallel polarization configuration at 20 K for ambient pressure and 2.5 GPa, respectively. c) and d) Corresponding polar plots of the Raman intensities of modes A, B, C, and G at ambient pressure and 2.5 GPa.

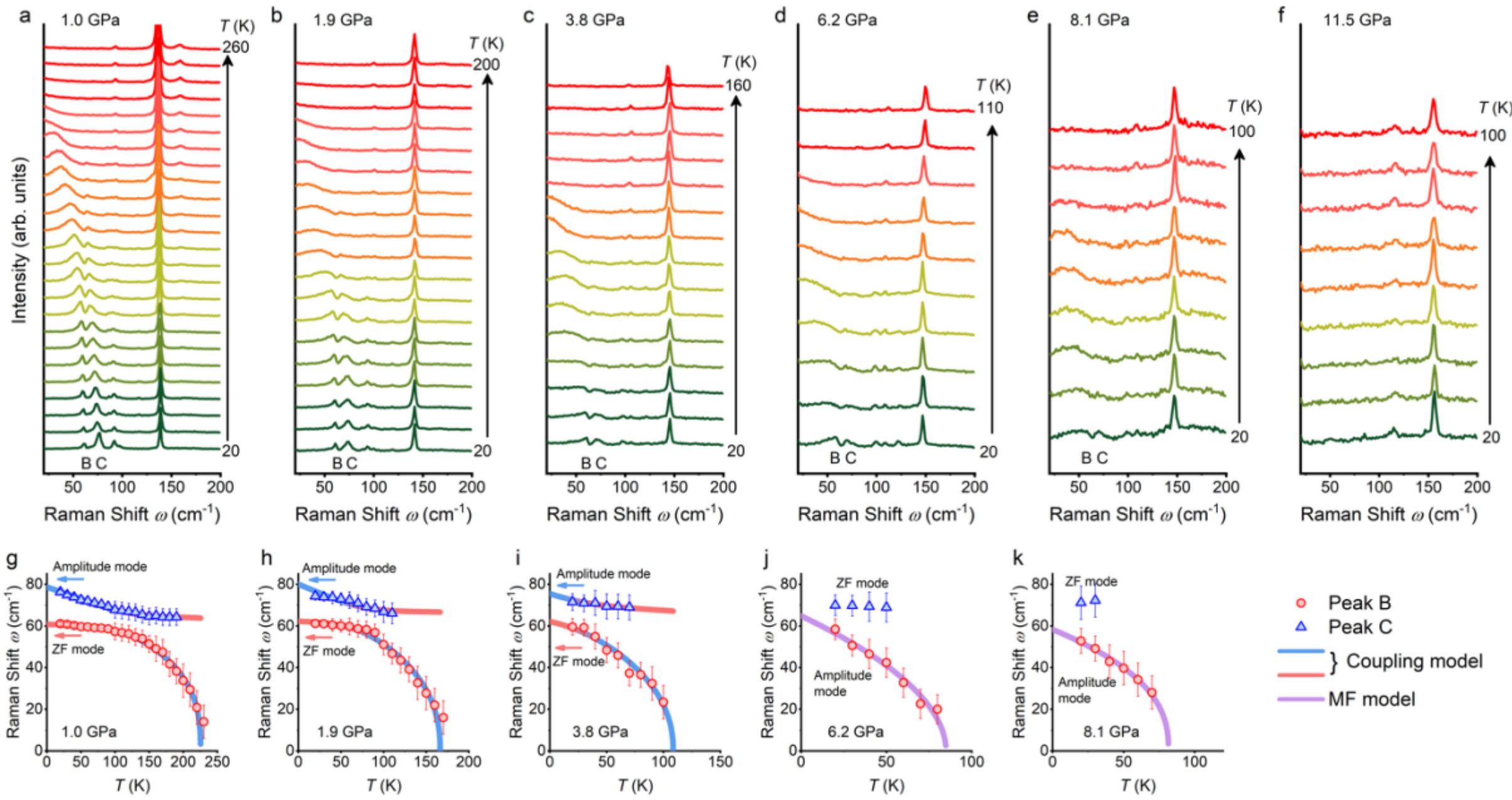

Figure S7. a) to f) Temperature-dependent Raman spectra under different pressures. g) to k) Temperature evolution of the Raman shifts of modes B and C. Solid curves denote fits using the coupling model for 1.0, 1.9, and 3.8 GPa. At 6.2 and 8.1 GPa, the anti-crossing effect between modes B and C is absent; in this pressure regime, mode B is described by a mean-field fit. At 11.5 GPa, no amplitude mode is observed, indicating complete suppression of the CDW state.

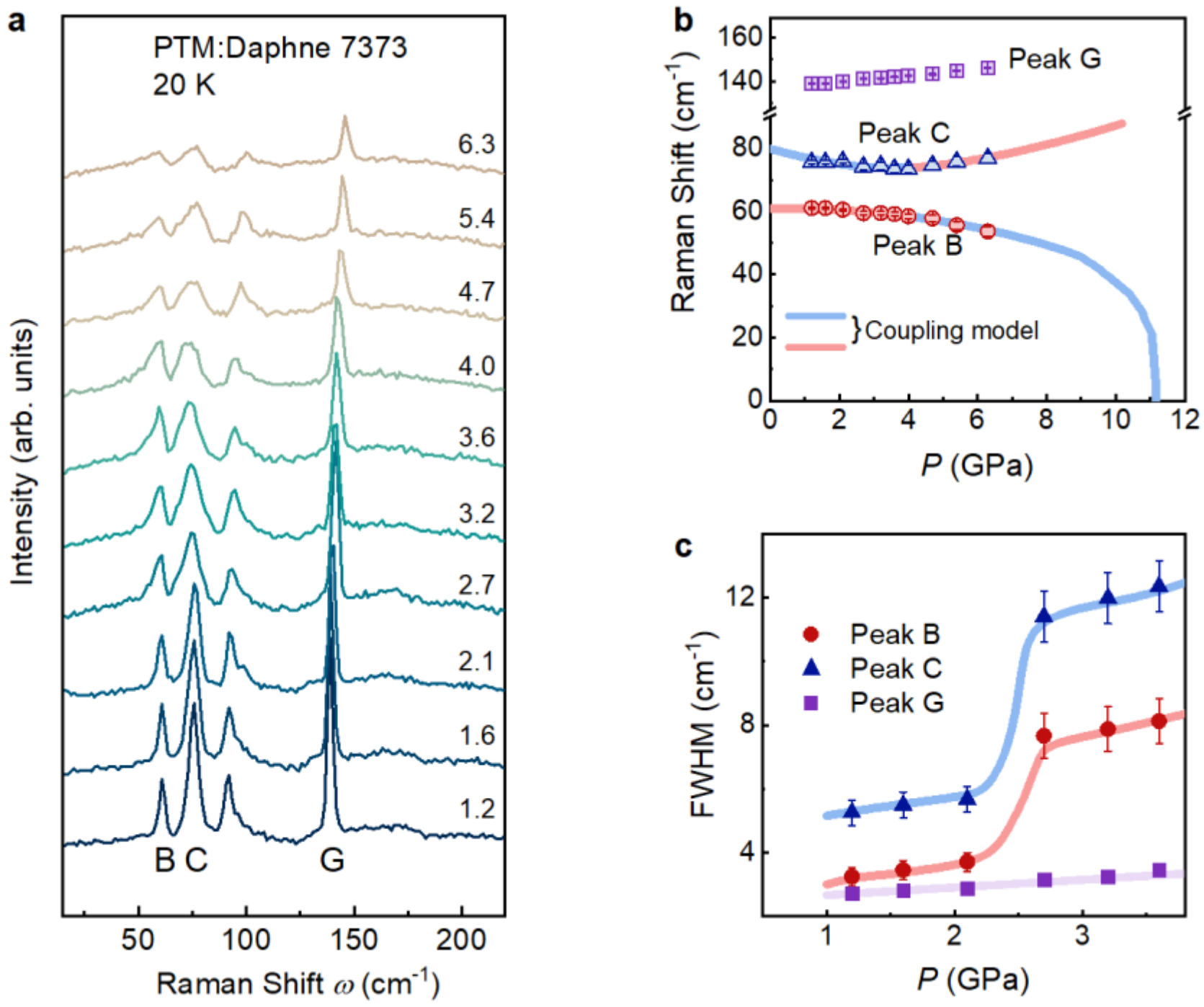

Figure S8. a) High-pressure Raman spectra measured at 20 K using Daphne 7373 oil as the pressure-transmitting medium (PTM). b) Pressure evolution of the Raman shifts of peaks B, C, and G. The solid curves represent fits to the coupled-mode model. c) Pressure dependence of the full width at half maximum (FWHM) of peaks B, C, and G.

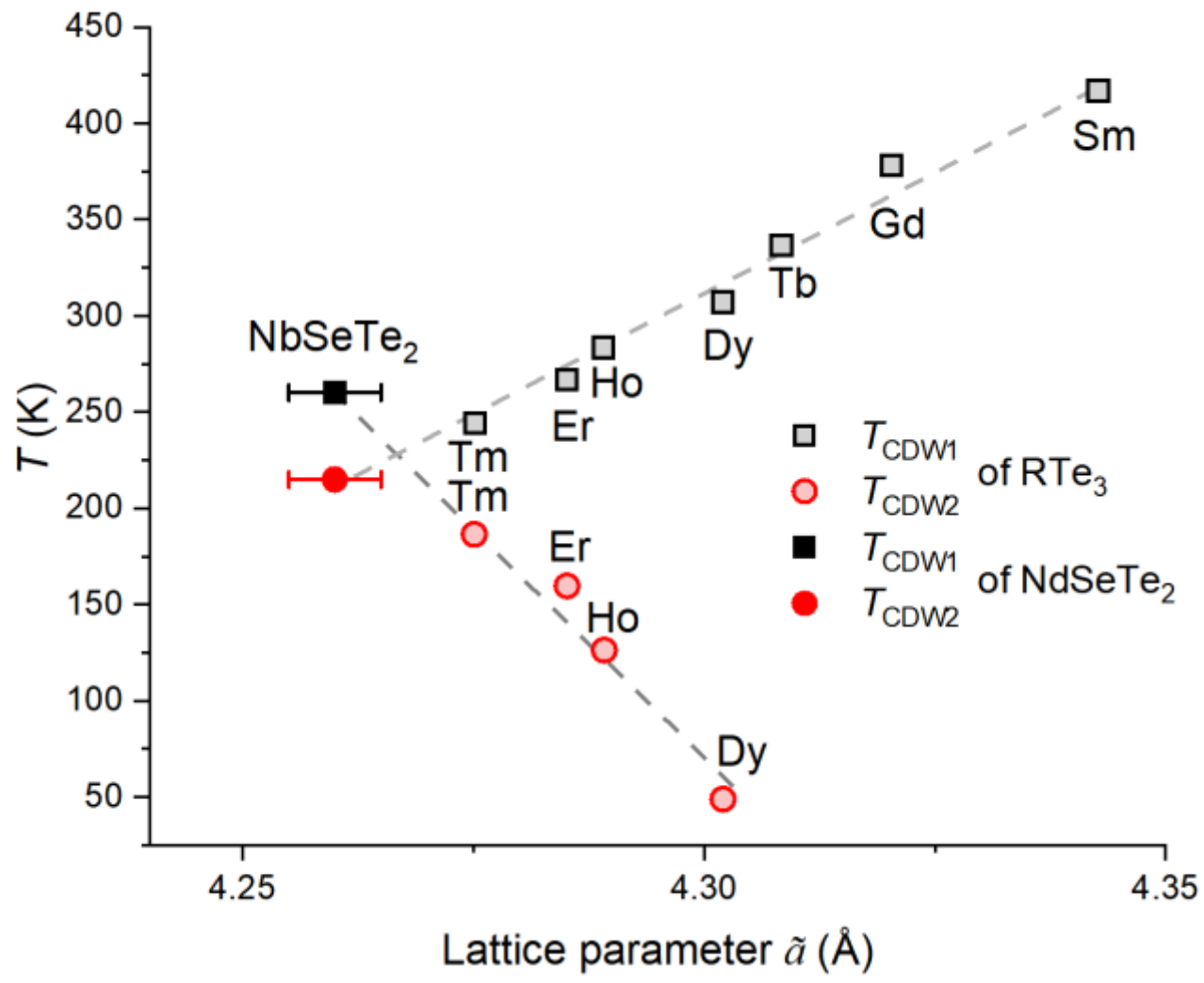

Figure S9. Ambient-pressure charge-density-wave transition temperature as a function of the average lattice parameter $\tilde{a}$ for several RTe_3 compounds [8] and for $NdSeTe_2$.

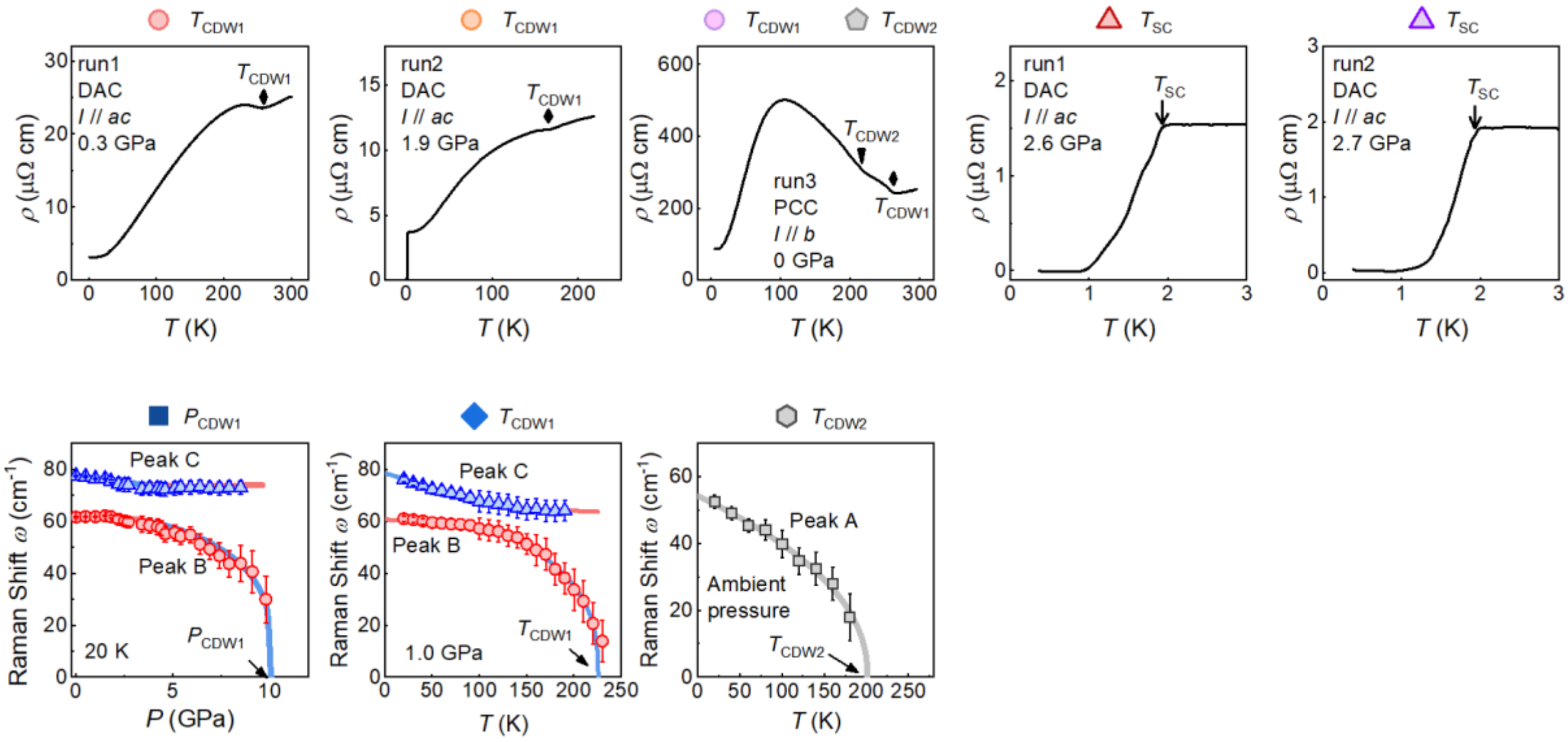

Figure S10. Definitions of the criteria used to determine each symbol in the phase diagram.

Note S3: High-pressure XRD measurements

To track the structural evolution under pressure, we carried out *in situ* high-pressure synchrotron XRD measurements on powdered single crystals of $NdSeTe_2$. As shown in Figure S10a, all diffraction peaks shift smoothly to higher angles without the emergence of new reflections up to 32.4 GPa. Using the Le Bail method, all XRD patterns remain indexed within the *Cmcm* space group. A representative fit at 0.2 GPa is displayed at the bottom of Figure S11a. All lattice parameters ($a = c$, b) decrease monotonically with pressure Figure S11b. The pressure dependence of the unit cell volume is well described by the third-order Birch-Murnaghan equation of state (solid red line in Figure S11c), yielding the ambient-pressure volume V_0 = 459.9 ± 1.4 Å^3, bulk modulus B_0 = 50.9 ± 1.6 GPa, and its first-order derivative B_0' = 5.3 ± 0.3.

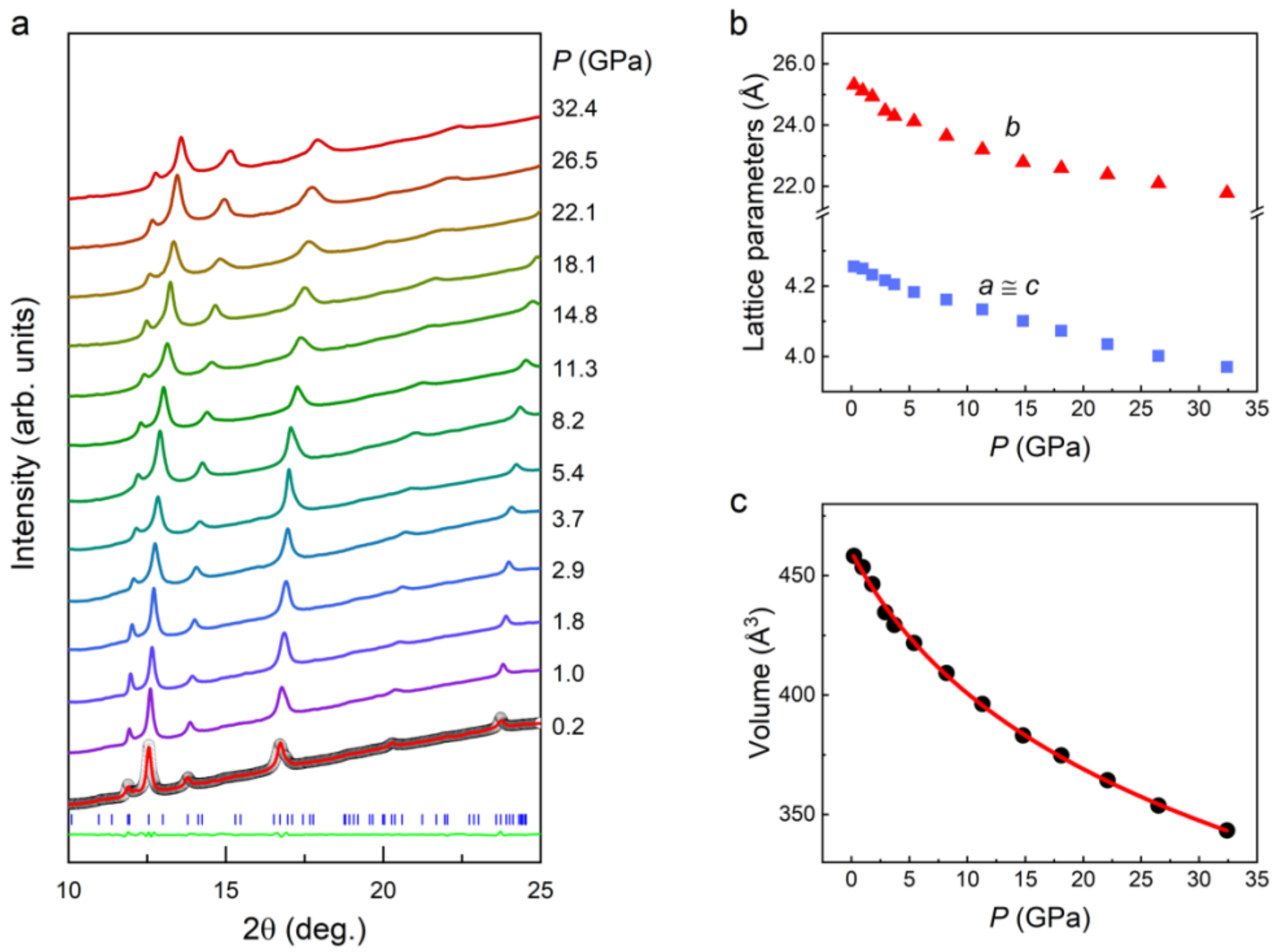

Figure S11. a) Pressure-dependent XRD patterns of $NdSeTe_2$ at room temperature (λ = 0.6199 Å). Bottom of (a): representative Le Bail refinement of the XRD pattern; vertical ticks mark the Bragg-peak positions, and the green line shows the difference between the measured and calculated data. b) Lattice parameters as a function of pressure. c) Unit-cell volume versus pressure, with the solid red curve representing a fit to the third-order Birch-Murnaghan equation of state.

Reference

1. R. Okuma, D. Ueta, S. Kuniyoshi, Y. Fujisawa, B. Smith, C. Hsu, Y. Inagaki, W. Si, T. Kawae, and H. Lin, "Fermionic order by disorder in a van der Waals antiferromagnet," *Sci. Rep.* **2020**, 10, 15311.
2. I. Berman, Z. Bynzarov, and Y. Kurkin, "Superconducting Properties of Te at Pressures Up to 260 Kbar," *Fizika Tverdogo Tela* **1972**, 14, 2527.
3. F. Bundy, and K. Dunn, "Pressure dependence of superconducting transition temperature of high-pressure metallic Te," *Phys. Rev. Lett.* **1980**, 44, 1623.
4. Y. Akahama, M. Kobayashi, and H. Kawamura, "Pressure-induced superconductivity and phase transition in selenium and tellurium," *Solid State Commun.* **1992**, 84, 803.
5. L. Zhao, C. Pei, J. Wu, Y. Zhao, Q. Wang, B. Zhu, C. Li, W. Cao, and Y. Qi, "Superconductivity and critical fields of tellurium single crystal under high pressure," *Phys. Rev. B* **2023**, 108, 214518.
6. N. Lazarević, Z. Popović, R. Hu, and C. Petrovic, "Evidence of coupling between phonons and charge-density waves in $ErTe_3$," *Phys. Rev. B* **2011**, 83, 024302.
7. B. Singh, G. McNamara, K.-M. Kim, S. Siddique, S. D. Funni, W. Zhang, X. Luo, P. Sakrikar, E. M. Kenney, and R. Singha, "Ferroaxial density wave from intertwined charge and orbital order in rare-earth tritellurides," *Nat. Phys.* **2025**, 21, 1578.
8. K. Yumigeta, Y. Qin, H. Li, M. Blei, Y. Attarde, C. Kopas, and S. Tongay, "Advances in rare-Earth tritelluride quantum materials: structure, properties, and synthesis," *Adv. Sci.* **2021**, 8, 2004762.
